# Distributional Variations in Trabecular Architecture of the Mandibular Bone: An *In Vivo* Micro-CT Analysis in Rats

**DOI:** 10.1371/journal.pone.0116194

**Published:** 2015-01-27

**Authors:** Zhongshuang Liu, Chengwei Yan, Chen kang, Bin Zhang, Ying Li

**Affiliations:** 1 Sino-Russian Institute of Hard Tissue Development and Regeneration, the Second Affiliated Hospital of Harbin Medical University, Harbin 150001, Heilongjiang, China; 2 Department of Pediatric Dentistry, the First Affiliated Hospital of Harbin Medical University, Harbin 150001, Heilongjiang, China; 3 Heilongjiang Academy of Medical Sciences, Harbin 150001, Heilongjiang, China; Oklahoma State University, UNITED STATES

## Abstract

**Purpose:**

To evaluate the effect of trabecular thickness and trabecular separation on modulating the trabecular architecture of the mandibular bone in ovariectomized rats.

**Materials and Methods:**

Fourteen 12-week-old adult female Wistar rats were divided into an ovariectomy group (OVX) and a sham-ovariectomy group (sham). Five months after the surgery, the mandibles from 14 rats (seven OVX and seven sham) were analyzed by micro-CT. Images of inter-radicular alveolar bone of the mandibular first molars underwent three-dimensional reconstruction and were analyzed.

**Results:**

Compared to the sham group, trabecular thickness in OVX alveolar bone decreased by 27% (P = 0.012), but trabecular separation in OVX alveolar bone increased by 59% (P = 0.005). A thickness and separation map showed that trabeculae of less than 100μm increased by 46%, whereas trabeculae of more than 200μm decreased by more than 40% in the OVX group compared to those in the sham group. Furthermore, the OVX separation of those trabecular of more than 200μm was 65% higher compared to the sham group. Bone mineral density (P = 0.028) and bone volume fraction (p = 0.001) were also significantly decreased in the OVX group compared to the sham group.

**Conclusions:**

Ovariectomy-induced bone loss in mandibular bone may be related to the distributional variations in trabecular thickness and separation which profoundly impact the modulation of the trabecular architecture.

## Introduction

Osteoporosis is a condition of low bone mass and microstructural changes in bone tissue, which leads to increased bone fragility and risk of fracture [1, 2, 3]. As the aging population increases, osteoporosis should be considered as a major growing public health problem. The most common and primary osteoporosis occurs in postmenopausal women, as a consequence of ovarian atrophy and the reduction of estrogen levels. This condition causes an imbalance between bone formation and resorption and leads to bone loss [4]. Bone remodeling is a lifelong process involving bone formation (mediated by osteoblasts) and bone resorption (mediated by osteoclasts). Imbalance between bone formation and resorption leads to metabolic bone diseases, such as osteoporosis [5]. In recent years, many studies have demonstrated the relationship between systemic osteoporosis and the loss of mandibular bone mass [6, 7, 8]. Ovariectomized rats have been widely used as experimental model for the study of postmenopausal osteoporosis in the spine and long bones [9, 10, 11]. However, studies on the relationship between bone loss in the jaw and estrogen deficiency are still controversial. Wende et al. indicated that osteoporosis is correlated with alveolar bone loss in postmenopausal women [12]. Kribbs reported that osteoporotic women had less mandibular bone mass and density compared to healthy women [13]. In addition, Tanaka et al. suggested that ovariectomy causes greatly accelerated bone resorption in the mandibular alveolar bone [14]. In contrast, Moriya et al. demonstrated that only ovariectomy may not be capable of causing periodontal destruction and alveolar bone loss [15].

Based on the studies above, understanding the influence of ovarian hormone deficiency on bone remodeling and bone structural changes of the mandible is needed. The efficacy of various measures for the assessment of trabecular bone architecture has been widely studied. Until recently, quantitative histologic techniques were the standard of the evaluation of trabecular and cortical bone architecture. The standard method to measure trabecular structure is histomorphometry of thin stained sections. Although histological analyses provide unique information on cell morphology and dynamic index of bone remodeling, this technique has several major limitations: it is destructive, the sample preparation is tedious and it is essentially 2D by nature [16]. However，microcomputed tomography imaging (micro-CT) is a powerful tool for quantifying bone quality of small samples non-destructively [17, 18]. With non-invasive 3D imaging techniques, trabecular bone morphology parameters were commonly denoted by an asterisk like mean trabecular thickness (Tb.Th*), and mean trabecular separation (Tb.Sp*) [19, 20, 21, 22]. But all these studies failed to show the distributions of the trabecular thickness and separation in the inter-radicular alveolar bone. In our study, thickness and separation maps of the inter-radicular alveolar bone were generated by Scanco Medical image processing software.

The present study used micro-CT to evaluate the effect of trabecular thickness and trabecular separation on modulating the trabecular architecture of the mandibular bone in ovariectomized rats.

## Materials and Methods

Fourteen 12-week-old adult female Wistar rats (approximate weight, 230–260 g) were used for this study. All the experimental protocols followed had been approved by the Ethics Committee of Harbin Medical University, and the experiments were carried out under the control of the University’s Guidelines for Animal Experimentation.

### Ovariectomy

The animals were divided randomly, seven were subjected to bilateral ovariectomy (OVX group), while the other 7 rats were selected for sham ovariectomy (sham group). All animals were housed under the same conditions and fed a standard Rodent diet with free access to water for 5 months.

### Micro-CT scanning and alveolar bone analysis

Five months after surgery, all rats were euthanized with an overdose of chloral hydrate and the mandibles were extracted and stored in 4% paraformaldehyde. Fourteen samples (seven OVX and seven sham) were scanned by a micro-CT scanner (μCT35, Scanco Medical AG, Bassersdorf, Switzerland) with a 12μm voxel size using the following parameters: 114mA, 70kVp, and exposure time of 300ms. The scanning procedure lasted about 1h per sample and generated approximately 600 images.

**Fig 1:**
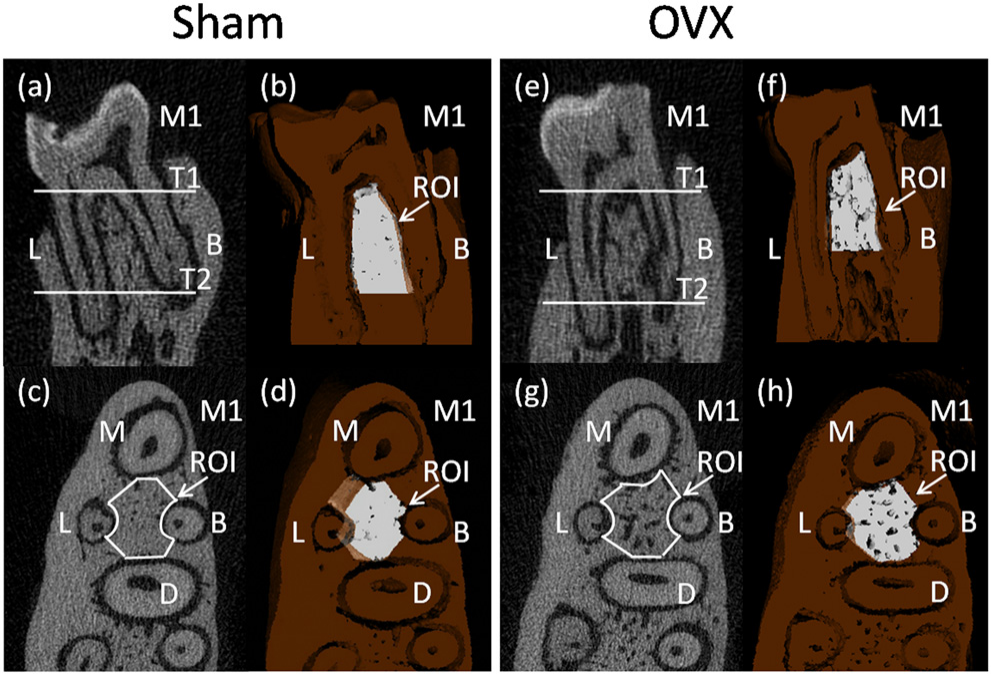
Micro-CT images of the rat mandibular alveolar bone and the mandibular first molar. a,b,c,d were obtained from a sham rat, while e,f,g,h were obtained from an ovariectomied rat. a,b,e,f showed the coronal surface passing the centers of the buccal root and the lingual root. c,d,g,h showed the horizontal surface which was parallel to the occlusion plane. M1, the mandibular first molar; M, mesial root; B, buccal root; D, distal root; L, lingual root. T1 and T2, two horizontal surfaces passed the alveolar ridge crest and apex of the buccal root separately and were parallel to the occlusion plane. a,c,e,g showed two-dimensional images, b,d,f,h showed three-dimensional images. In the 3D reconstruction images, the deep brown part represented alveolar bone and the white part represented the region of interest (ROI).

The region of interest (ROI) of the alveolar bone was performed according to previously described method which succeeded in showing the integral structure of alveolar bone [19]. The ROI of the alveolar bone was manually established in the inter-radicular septal bone of the left mandibular first molar (M1), as there is an obvious stress concentration in this region which is frequently used for histomorphometry of trabecular bone. First, we identified the coronal surface passing the centers of the buccal root and the lingual root on two-dimensional images (Fig. 1a,e). Then, we chose two horizontal surfaces passing the alveolar ridge crest and apex of the buccal root separately and they were parallel to the occlusion plane (T1 and T2 in Fig. 1a,e). The white part showed the upper and lower borders of the ROI on three-dimensional images (Fig. 1b,f). Third, we selected out the inter-radicular alveolar bone by drawing a contour from the center of one root canal to another, avoiding roots and other structures on two-dimensional image which showed a horizontal plane of mandibular first molar (Fig. 1c,g). The white part showed the ROI between the centers of the four roots in the M1 on the horizontal surfaces (Fig. 1d,h).

Three-dimensional microarchitecture of the alveolar bone was analyzed by the software affiliated to the micro-CT. The alveolar bone histomorphometry were calculated directly from the ROI. We evaluated the bone volume/total volume (BV/TV,%), trabecular thickness (Tb.Th, mm), trabecular number (Tb.N. per mm), trabecular separation (Tb.Sp, mm), and bone mineral density (BMD).

### X-ray

**Fig 2:**
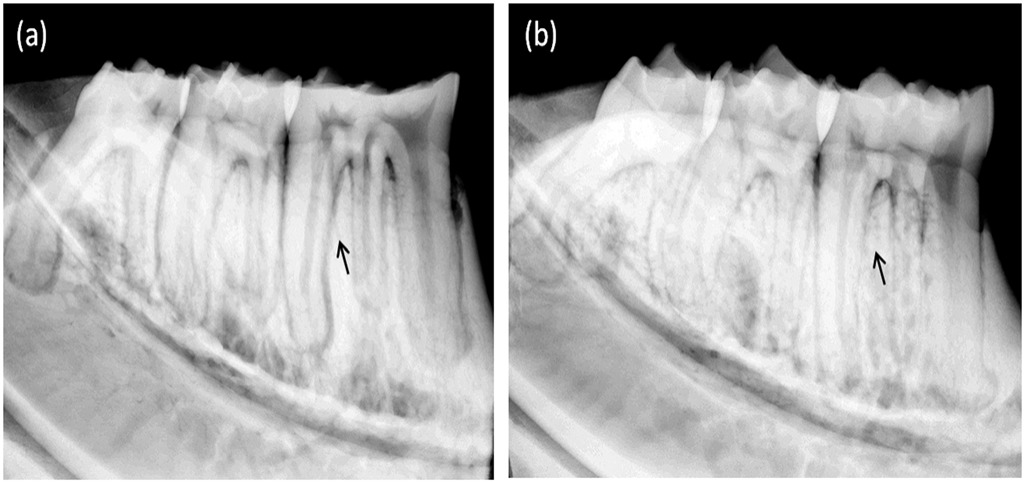
Analysis of mandible on radiographs: (a) Sham group (b) OVX group. The arrows showed the inter-radicular alveolar bone loss of the molars.

The lingual side of left mandibular bone were radiographed (Fig. 2) by a Faxitron Specimen Radiography System (Model MX-20; Faxitron X-ray Corporation, Wheeling, IL) at 26 kVp, exposure-time of 8.5s.

### Statistical analysis

All data was presented as means±SD, and statistical analysis was carried out by using SPSS, version 13.0 (SPSS Inc., Chicago, IL, USA). Parameters of the sham and OVX groups were compared by using the t-test. A result was considered as statistically significant at P < 0.05.

## Results

### Micro-CT histomorphometry of alveolar bone structure analysis

**Fig 3:**
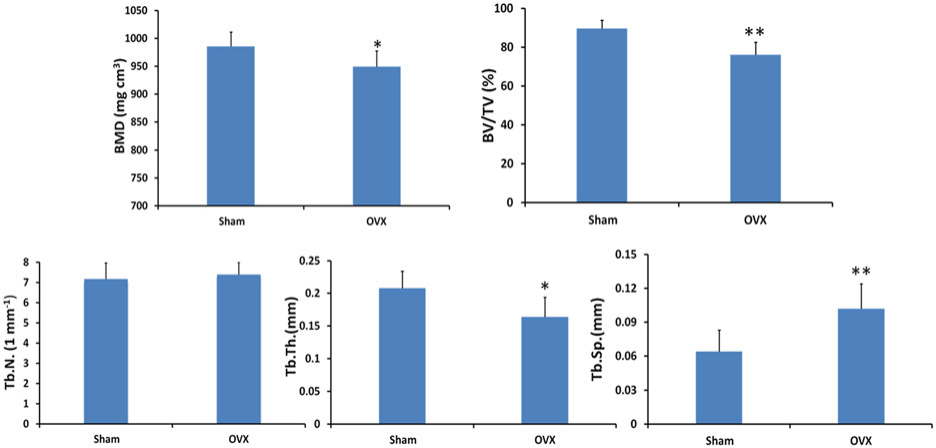
Micro-tomographic histomorphometric comparisons between the sham and OVX groups. Values are mean±S.D. Significant differences between the two groups were marked with asterisks (*P < 0.05; **P < 0.01).

Micro-CT was able to assess trabecular bone morphology of alveolar bone in both two-dimensional and three-dimensional views. Two-dimensional images of coronal sections (Fig. 1a,e) and horizontal sections (Fig. 1c,g) showed the marrow spaces, in which the inter-radicular alveolar bone encompassing mandibular first molar in the OVX group increased as compared with that in the sham group. Three-dimensional images obtained from micro-CT scanning were used to visualize the region of the inter-radicular alveolar bone in rats (Fig. 1b,d,f,h). The quantification of micro-CT trabecular bone changes (mean±SD) was shown in the OVX and sham groups (Table 1 and Fig. 3). All trabecular parameters in alveolar bone showed significant changes. The inter-radicular alveolar bone trabecular bone volume fraction (BV/TV) was found to be lower in the animals of the OVX group as compared to those of the sham groups (p = 0.001). Moreover, lower bone mass and looser construction of more widely separated trabeculae could be observed in OVX rats. At the same time, BMD was significantly reduced in the OVX rats compared to that of the sham rats (P = 0.028). Furthermore, the inter-radicular alveolar bone loss of the molars was confirmed through X-ray measurement between two groups in Fig. 2. However, trabecular number exhibited no significant differences either in sham or in OVX rats.

**Table 1:** The microarchitectural parametric values of the mandibular bone in the inter-radicular region (n = 7).

	**Sham**	**OVX**	***P***
BV/TV (%)	89.71±4.10	76.13±6.52	0.001**
Tb.N.(1 mm^-1^)	7.174±0.80	7.398±0.61	0.564
Tb.Th. (mm)	0.208±0.026	0.164±0.030	0.012*
Tb.Sp. (mm)	0.064±0.019	0.102±0.022	0.005**
BMD (mg cm^3^)	985.6±25.89	949.35±28.38	0.028*

BV/TV, bone volume fraction; Tb.N., trabecular number; Tb.Th., trabecular thickness; Tb.Sp., trabecular separation; BMD, bone mineral density.

* *P* < 0.05,

** *P*< 0.01.

### Trabecular thickness and trabecular separation analysis

**Fig 4:**
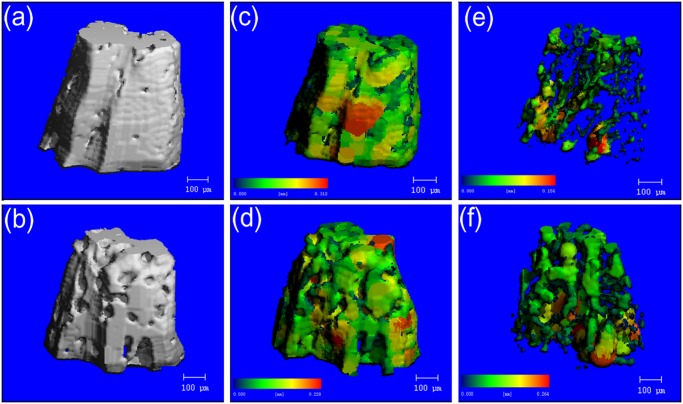
Representative image illustrating micro-CT image analysis in the inter-radicular region of the mandibular bone. a, c, e were obtained from a sham rat, while b, d, f were obtained from an ovariectomied rat. Pseudocolor coding displayed larger thicknesses and seperations in red and smaller thicknesses and seperations in blue/green. a,b showed the 3D reconstruction images. The thickness map (c,d) and the separation map (e,f) generated after segmentation of teeth and bone demonstrated the ability to quantitatively analyze trabecular bone morphology.

Images of inter-radicular alveolar bone generated by micro-CT were shown in Fig. 4. a and b showed the 3D reconstruction images. Pseudocolor coding displayed larger thicknesses and seperations in red and smaller thicknesses and seperations in blue/green. The thickness map (c,d) and the separation map (e,f) generated after segmentation of teeth and bone demonstrated the ability to quantitatively analyze trabecular bone morphology.

**Fig 5:**
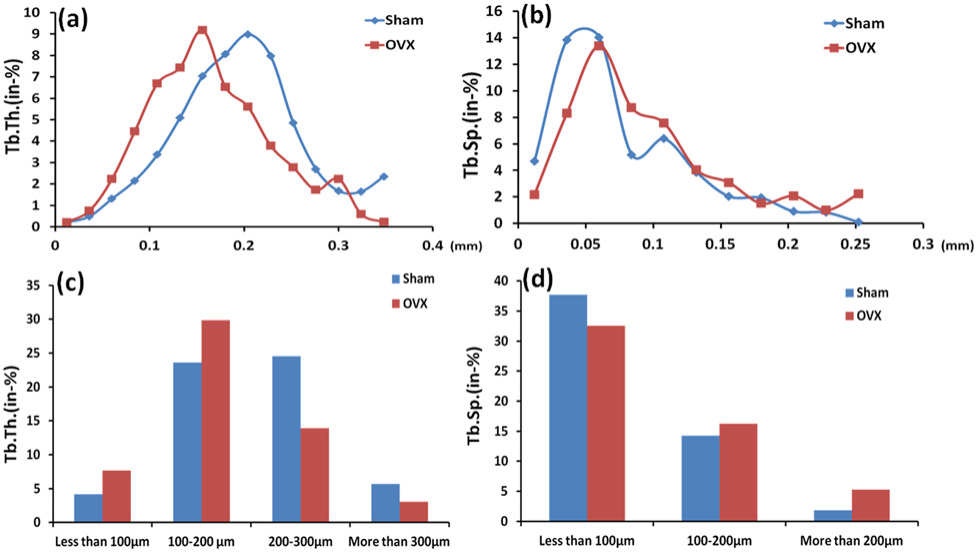
Comparison of the distributions of the trabecular thickness (a) and the trabecular separation (b) in the inter-radicular region of the mandibular first molar between the Sham and OVX groups; Comparison between two groups of the different ranges for trabecular thickness (c) and trabecular separation (d). Tb.Th., trabecular thickness; Tb.Sp., trabecular separation.

Trabecular thickness in OVX alveolar bone decreased by 27% (P = 0.012) compared to the sham group. Trabecular separation in OVX alveolar bone increased by 59% (P = 0.005) compared to the sham group (Fig. 3). Compared with the compact structure of alveolar bone in sham rats, there were more pores on the surfaces of alveolar bone in OVX rats (Fig. 4a,b). After segmentation of inter-radicular alveolar bone from jaw bone, the thickness map (Fig. 4c,d) and separation map (Fig. 4e,f) of the inter-radicular alveolar bone surface layer indicated significant differences in Tb.Th. and Tb.Sp. distribution of the alveolar bone between the sham and OVX rats. Tables 2 and 3 showed comparision of the distributions of the trabecular thickness and trabecular separation in the inter-radicular region of the mandibular bone between the sham and OVX groups. It provided the percentage of trabeculae with different thicknesses and trabecular separation (In-%) with an assessment through the software affiliated to the micro-CT in the sham and OVX groups. Obvious differences in trabecular thickness and separation between the OVX rats and the sham rats could be found in Fig. 5.

**Table 2:** Comparison of the distributions of the trabecular thickness in the inter-radicular region of the mandibular bone between the sham and OVX groups.

Tb.Th.Group	0.012	0.036	0.06	0.084	0.108	0.132	0.156	0.18	0.204	0.228	0.252	0.276	0.300	0.324	0.348
Sham(in-%)	0.197	0.485	1.311	2.152	3.372	5.101	7.038	8.066	8.999	7.978	4.856	2.696	1.672	1.647	2.353
OVX(in-%)	0.224	0.752	2.247	4.453	6.693	7.446	9.184	6.537	5.608	3.791	2.783	1.731	2.246	0.598	0.229

Tb.Th., trabecular thickness (mm).

Sham (in-%) and OVX (in-%) respectively represent the percentages of trabeculae with different thicknesses in the sham and OVX groups.

**Table 3:** Comparison of the distributions of the trabecular separation in the inter-radicular region of the mandibular bone between the sham and OVX groups.

Tb.Sp.Group	0.012	0.036	0.060	0.084	0.108	0.132	0.156	0.18	0.204	0.228	0.252
Sham (in-%)	4.675	13.838	14.018	5.183	6.387	3.863	2.045	1.933	0.913	0.828	0.109
OVX (in-%)	2.175	8.300	13.374	8.719	7.566	4.052	3.088	1.517	2.070	1.009	2.222

Tb.Sp., trabecular separation (mm).

Sham (in-%) and OVX (in-%) respectively represent the percentages of trabecular separation in the sham and OVX groups.

Trabeculae of less than 100μm and 100–200μm in the OVX group increased by 46% and 21% respectively compared to those in the sham group. Trabeculae of 200–300 μm and more than 300μm in the OVX group were 43% and 46% lower than those in the sham group. Furthermore, trabecular separation of less than 100μm in the sham group was 16% higher than the OVX group. The OVX group had significantly higher trabecular separation by 14% and 65% for trabecular separation of 100–200μm and more than 200μm, respectively, compared to the sham group. At the same time, trabeculae of 156μm thick was dominant in the alveolar bone in the OVX group, and the same thickness in sham rats was about 23% lower than that in OVX rats. However, trabeculae of 204μm thick was dominant in the alveolar bone in the sham group, and the same thickness in sham rats was about 38% higher than that in OVX rats. In addition, trabecular separation of 60μm took over the majority in the alveolar bone in both the sham and OVX groups, but trabecular separation of more than 60μm in the OVX group was almost always significantly higher than that in the sham group. Specifically, the difference reached more than 30% between the two groups (Fig. 5).

## Discussion

Osteoporosis is becoming a public health problem because this disease appears in the elderly, especially postmenopausal women. Estrogen deficiency would be expected to increase bone resorption and impair bone formation [23, 24]. With the increasing number of elderly dental patients, the effects of osteoporosis on alveolar bone have become an important issue in the field of dentistry. Previous studies showed that osteoporosis may significantly affect the alveolar bone remodeling processes, which have been observed in treatments such as orthodontic movement, implant restoration, periodontal treatment [25]. Micro-CT systems have become an effective and noninvasive method for the assessment of bone microstructural features. The advantage of using micro-CT is that it can show internal and external alveolar bone anatomy in smallest possible fraction clearly and accurately [17, 26]. The efficacy of various measures by micro-CT for the assessment of trabecular bone architecture has been widely studied, but all these methods failed to show the distributions of the trabecular thickness and separation in the inter-radicular alveolar bone of the mandible [19, 27]. In our study, thickness and separation maps of the inter-radicular alveolar bone were generated by using Scanco Medical image processing software. Larger trabecular thickness could be observed in the sham group compared to the OVX group. Moreover, trabecular thickness of less than 200μm significantly increased in the OVX rats compared to the sham rats. Correspondingly, the OVX group had significantly higher trabecular separation for trabecular separation of more than 200μm compared to the sham group. According to the significant variations between the sham and OVX rats, it explained that a looser structure of more widely separated trabeculae could be observed in OVX rats. Compared to other researches, our study not only showed the mean trabecular thickness and mean trabecular separation to indicate the effect of osteoporosis on the bone quality and quantities, but also exhibited significant difference in the distribution of trabecular thickness and separation between OVX and sham rats [28, 29, 30, 31].

Postmenopausal osteoporosis is a kind of metabolic osteopathy that produces bone of lower quality, and osteoporosis of mandible often leads to the failure of dental prosthetics [28]. Bone micro-architecture is an important factor, which contributes to bone quality. Obviously, the distribution of trabecular thickness and separation has effects on the alveolar bone mechanical property, which is very important for the stability and long-term success of dental treatment in osteoporosis. Because the mean trabecular thickness and separation are the most important parameters to estimate the microstructure of cancellous bone, some studies have indicated that trabecular thickness and separation are necessary for reliable investigation of morphologic changes in bone [17, 19]. However, even if the elderly dental patients have the similar mean trabecular thickness and separation, it still wouldn’t prove that their bone is in approximately the same structure. Therefore, establishing a proper evaluation method is very important to understand trabecular architecture under osteoporotic situation. In this paper, a new method was proposed to evaluate the effect of trabecular thickness and separation on modulating the trabecular architecture of the mandibular bone in ovariectomized rats.

Furthermore, the present study indicated that the peak value of trabecular thickness in the inter-radicular alveolar bone was obviously decreased from 204μm in the sham rats to 156μm in the OVX rats. The values of the trabecular thickness in the OVX group were considerably lower than those in the sham group. Specifically, the difference reached more than 30% between the two groups. In addition, trabecular separation of more than 60 μm significantly increased in the OVX rats compared to the sham rats. The value of trabecular separation in the sham group was approximately 21%, which was about 10% lower than that of the OVX group. These differences may be a consequence of the internal organization structures of trabecular bone changing in ovariectomized rats [15, 28]. Considering the influences of osteoporosis, the analysis revealed an expected significant difference in the peak value of trabecular thickness and the distribution of trabecular separation of more than 60μm. Despite the great impact caused by osteoporosis, some investigators continued to use mean value analysis to trabecular structural parameters without considering the effect of distributional variations in trabecular architecture on their evaluation [32, 33]. Therefore, it is important to take distribution of trabecular thickness and separation of alveolar bone into account so that the trabecular architecture can be analyzed accurately.

There was also a significant decrease of bone volume fraction, bone mineral density confirmed by micro-CT analysis in the OVX rats when compared with the sham group. The OVX condition resulted in alteration of the trabecular distance and thickness as compared to the sham group, which indicated profound impact on the histological integrity of the bone. The reduction in trabecular bone volume accompanied by the expansion of bone marrow area in the alveolar bone encompassing mandibular molars was in agreement with another investigation [19]. In this context, these results may describe a state of osteopenia in the OVX rats, which might be the result of an imbalance between bone formation and resorption. The effects of estrogen-deficiency on the bones were complex. It was mentioned in literature that estrogen exerts direct anabolic effects on bone cells [34]. It stimulated bone resorption or bone formation and regulated osteoclastic differentiation from hematopoietic mononuclear cells, and osteoblastic functions and activity. Tanaka et al previously reported high bone turnover of mandibular alveolar bone in OVX rats, in both high osteoclastic and osteoblastic parameters [14]. Moreover, osteoclasts showed reduced bone resorption in response to ovarian estrogen-deficiency [24]. These findings support the idea that estrogen-deficiency in bone could directly mediate the stimulation of osteoblasts and inhibition of osteoclasts, which may be the reason for the porotic changes in trabecular structure. Furthermore, it could have profound effects on biomechanical behavior of bone.

BMDs were continually evaluated for 5 months after the ovariectomy. Analysis of mandibular BMD data revealed that the sham group and OVX group had significant differences in statistics. This was comparable to the results of Rawlinson et al., which showed that the period of 5 months was sufficient to detect statistical changes in the mandibular bone structure [35]. The BMD in the OVX group was lower (P <0.001) than that in the sham group. Results relative to the OVX group agreed with other studies [9, 36, 37, 38] that found lower BMD after ovariectomy. Contrary to the results presented here, Kuroda et al. showed no differences between the sham and OVX groups for the inter-radicular region of the first molar after OVX as evaluated by DXA [39].

Consider that rat’s incisors occupy a large area, it may change the DXA analyses because the mineral content of incisors and molars is included in global BMD evaluation. Removal of these teeth may change the mandible bone structure, while teeth extraction for the evaluation of DXA was a more complex process, particularly in the region near dental elements which may limit or alter the BMD read by DXA [40]. Micro-CT has been used for a wide range of studies to estimate bone mineral density [41, 42]. Compared to DXA, the advantage of using micro-CT for assessment of bone mass was that the ROI can be performed as required [17, 43, 44]. In our study, the ROI of the alveolar bone was successfully performed while avoiding the influence from the molars. Consequently, the degree of mineralization in the inter-radicular alveolar bone can be analyzed accurately by micro-CT.

The results of the present study seem to confirm the hypothesis that estrogen levels have an important effect on alveolar bone. Significant adaptation of trabecular bone architecture in alveolar bone to a modified OVX environment happened. This, extrapolated to humans, could mean that for those elderly postmenopausal women who seek dental treatment, the effect of postmenopausal osteoporosis on the alveolar bone quantity and quality can be expected. However, the micro-CT analysis technique cannot, at present, be used for dynamic bone histomorphometry. Furthermore, the measurement of the dynamics of bone formation is needed for further studies to evaluate the influence of estrogen deficiency on the alveolar bone.
